# Inference of demographic history from genealogical trees using reversible jump Markov chain Monte Carlo

**DOI:** 10.1186/1471-2148-5-6

**Published:** 2005-01-21

**Authors:** Rainer Opgen-Rhein, Ludwig Fahrmeir, Korbinian Strimmer

**Affiliations:** 1 Department of Statistics, University of Munich, Ludwigstr. 33, D-80539 Munich, Germany

## Abstract

**Background:**

Coalescent theory is a general framework to model genetic variation in a population. Specifically, it allows inference about population parameters from sampled DNA sequences. However, most currently employed variants of coalescent theory only consider very simple demographic scenarios of population size changes, such as exponential growth.

**Results:**

Here we develop a coalescent approach that allows Bayesian non-parametric estimation of the demographic history using genealogies reconstructed from sampled DNA sequences. In this framework inference and model selection is done using reversible jump Markov chain Monte Carlo (MCMC). This method is computationally efficient and overcomes the limitations of related non-parametric approaches such as the skyline plot. We validate the approach using simulated data. Subsequently, we reanalyze HIV-1 sequence data from Central Africa and Hepatitis C virus (HCV) data from Egypt.

**Conclusions:**

The new method provides a Bayesian procedure for non-parametric estimation of the demographic history. By construction it additionally provides confidence limits and may be used jointly with other MCMC-based coalescent approaches.

## Background

The coalescent is a very versatile stochastic model of the genetic variation in a set of sequences sampled from a population. It allows to accommodate a wide range of assumptions about rates and modes of evolution, and of population history [1-5].

As the observed sequence data are positively correlated due to common ancestry, coalescent theory also provides a framework for understanding the relationship between a population's history and its genealogy. For instance, it has long been noted that genealogies of samples taken from exponentially growing populations tend to be "star-like" with short branch lengths near the root of the tree. In contrast, the inter-node distances in genealogies from constant-size populations typically are much more evenly spaced. 

Thus, coalescent theory quantifies the imprint that demographic development of a population leaves in the data. While the original theory was outlined for constant population size [1,2], Slatkin and Hudson [6] soon developed a coalescent model for the case of an exponentially growing population. Subsequently, a general approach allowing arbitrary population size variation through time was presented by Griffith and Tavaré [7].

Therefore at least in principle the coalescent model provides a basis for *statistically inferring the demographic history *as a function of time from the sampled sequences [3,8-12] or, alternatively, from the corresponding inferred genealogies [13-15]. In practice, however, application of coalescent theory to this problem has been restricted to very simple demographic scenarios such as constant size, exponential or logistic growth.

Only recently methods have emerged that attempt the completely non-parametric estimation of the demographic function from the data. Polanski et al. proposed an approach based on pairwise distances [16], hence generalizing the method by Slatkin and Hudson [6]. Pybus et al. [14] presented the "skyline plot" method that uses a step-function to approximate the population history obtained from an estimated genealogy. This method was subsequently refined to the "generalized skyline plot" [17] which is essentially a regularized version of the classic skyline plot. If the population size is truly constant through time the generalized skyline plot estimate of population size collapses to the phylogenetic coalescent estimator proposed by Felsenstein [13].

The advantage of the skyline plot over the method suggested by Polanski et al. [16] is that it takes into account the genealogical relationship among the sequences. This helps to decrease bias and improves the efficiency of the resulting estimator compared to methods based on summary statistics and pairwise distances [13]. Unfortunately, the skyline plot approach also has several deficiencies. First, it is unclear how to extend the approach to allow multiple genealogies as input. This is important in order to accommodate phylogenetic error, and to allow non-parametric inference of population history in coalescent approaches that take all possible genealogies into account [7-10]. Second, and perhaps more critical, the (generalized) skyline plot only provides a population size trend rather than a realistic estimate of population size changes, as by construction the population function is modeled by a step function. Moreover, the change-points of this function are fixed at the inter-nodes of the underlying tree.

In this paper we propose a novel framework to non-parametric estimation of the demographic history. This approach relies on Bayesian reversible-jump MCMC inference [18] to obtain a smooth population size function from a given set of genealogies. The new method not only renders many deficiencies of the classic and generalized skyline plot obsolete but it is also computationally efficient, with running times of the algorithm for typical data in the order of minutes on standard PC hardware. The framework has been implemented in the computer language R [19] and incorporated in the R package APE [20].

The remainder of the paper is organized as follows. In the next section we describe the mathematical and statistical theory of the new framework. Subsequently, we apply the method to simulated and biological sequence data and discuss the results. In the last section we briefly outline possible further extensions and related directions of research.

## Results

### Background in coalescent theory

#### Basic model

In a pan-mictic population with constant effective population size *N*_*e*_, where every individual has a single parent, the waiting time *w*_*n *_until any two of *n *sampled lineages coalesce is exponentially distributed with rate [1,2]. For *n *sequences there are therefore *n *- 1 intervals *I*_*n*_, *I*_*n*-1_,..., *I*_2 _with rates *r*_*n*_, *r*_*n*-1_,..., *r*_2 _and interval lengths *w*_*n*_, *w*_*n*-1_,..., *w*_2_. With  we denote the time until all samples have reached the most recent common ancestor.

The coalescent model implies that the waiting time to the next coalescent event follows an inhomogeneous Poisson-process with a hazard rate *r*_*n *_that varies in time *t *because of the change in the number of lineages. Thus, it is straightforward to also include variable population size in the coalescent simply by using the hazard rate . From standard theory in survival analysis [21] it follows that the corresponding density for the waiting times is given by



where *τ*_*i *_is the time at the beginning of the interval *I*_*i*_. This is exactly the distribution from the variable population size coalescent



as developed in [7]. The coalescent model can be further expanded to diploid populations [22] or to include other effects like selection, recombination or geographical structures [4]. In this paper, however, we focus solely on the coalescent/survival model given by Eq. 2.

#### Estimation of population size

If the waiting times *w*_*i *_are known Eq. 2 can be used directly to estimate *N*_*e*_(*t*). This is typically done by maximizing the likelihood  assuming a simple parametric model for the population size change. For constant population size this has been done in [13], for more complicated scenarios such as logistic growth see, e.g., [14].

In a typical setting, however, the waiting times are themselves estimated from sequence data. In this case the total likelihood function will be a weighted sum of the likelihoods for all possible waiting times, so that in effect the *w*_*i *_are marginalized out in favor of the actually observed data. In practice exact calculation of this sum is prohibitive, hence one relies on approximating MCMC methods [8-10].

As a shortcut to avoid these computationally very expensive procedures one may also substitute the "true" waiting times by those obtained from inter-node distances of a single estimated gene tree (see, e.g., [23] for an overview of relevant likelihood-based tree inference methods) and proceed as above. Note that the resulting plug-in approximation ignores the uncertainty from estimating the *w*_*i *_in the inference of demographic parameters. However, this is justifiable if the phylogenetic error is much smaller than the error introduced by the coalescent. This will be the case if sequences are sufficiently long and the substitution rate is comparatively high (a typical example would be virus data).

For non-parametric estimation of population size, Pybus et al. suggested the "skyline plot" [14]. This method assumes a piece-wise constant function for the population size *N*_*e*_(*t*) and allows population size changes only at the beginning and end of an interval *I*_*i*_. The estimated effective population size  in interval *I*_*i *_according to the skyline plot is given by the simple relation



**Figure 2:**
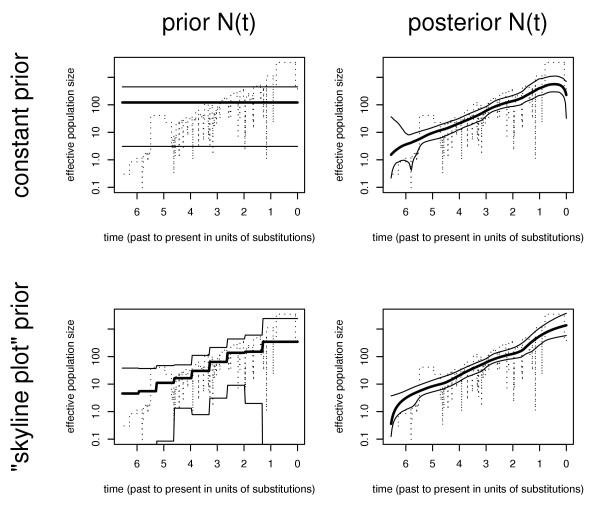
**Comparison of prior and posterior demographic functions ***Top row*: Bayesian inference using a prior demographic function with constant mean and constant variance (a 95% confidence band is indicated by showing the 2.5% and 97.5% quantiles). *Bottom row*: Bayesian inference using the "skyline plot" prior function.

This is the maximum likelihood estimate under the assumed model of fixed change-points. The "generalized skyline plot" subsequently introduced by Strimmer and Pybus [17] reduces the over-fitting present in the classic skyline plot by applying a simple form of regularization: adjacent intervals that alone are likely to have high stochastic noise are pooled together (cf. Fig. 2b and 2d). Choice of an optimal grouping of intervals (i.e. model selection) is performed by employing a second-order variant of the Akaike criterion [24].

### A Bayesian non-parametric approach to estimating demographic history

#### Outline

In this paper we present a non-parametric approach to infer population size changes in time that overcomes the limitations of previous approaches. More specifically, we develop a non-parametric Bayesian estimator for the function *N*_*e*_(*t*) conditioned on observed or sampled inter-node distances *w*_*n*_, *w*_*n*-1_,..., *w*_2 _by determining the posterior distribution P(*N*_*e*_(*t*)|*w*_*n*_, *w*_*n*-1_,..., *w*_2_). In order to sample the non-parametric demographic function from this posterior we use the reversible jump Markov chain Monte Carlo (rjMCMC) algorithm [18]. As a result, we obtain for any given time *t *both a point estimate  – here we choose the posterior median – as well as the associated credible interval (e.g., the lower and upper 2.5% quantiles). If the considered inter-node distances *w*_*n*_, *w*_*n*-1_,..., *w*_2 _are fixed and obtained from a single estimated tree, the resulting method is already directly applicable to phylogenetically informative data such as viral sequences (this is the focus of this paper). However, sampling of non-parametric demographic functions can also be combined in a conceptually straightforward fashion with sampling of trees, as outlined below.

#### Bayesian inference using reversible jump MCMC

In a nutshell, Bayesian inference of a parameter *x *consists of updating its prior distribution P(*x*) to a posterior distribution P(*x*|*D*) that takes account of the information in the observed data *D*. The relative evidence of the data for different values of *x *is summarized in the likelihood *L *= P(*D*|*x*) that accordingly plays a central role in the computation of the posterior via Bayes' theorem



For most realistic problems the posterior distribution cannot be computed analytically, in particular if *x *is a high-dimensional vector. Instead, one utilizes computational procedures to efficiently draw random samples from the posterior. This in turn allows computation of summary statistics such as the median or the upper and lower 2.5% quantiles. Markov chain Monte Carlo (MCMC) is one particularly useful sampling algorithm as it doesn't require calculation of the sum (or integral) in the nominator of Eq. 4. Briefly, sampling via MCMC is done by constructing a Markov chain with the possible combinations of parameter values as "states", and the desired posterior as its stationary distribution. These properties can be guaranteed by following certain rules for accepting or rejecting proposed new parameter values. Here we use the Metropolis-Hastings-Green method, i.e. the reversible jump MCMC algorithm [18], that has the advantage of not only allowing changes in the parameters values but also in the dimension of the parameter vector itself. Specifically, if *x *is the initial state, and  a proposed new state with proposal density , then the acceptance probability according to Green [18] is



where  is the likelihood ratio P(*D*|)/P(*D*|*x*),  is the prior ratio P()/P(*x*),  is the proposal ratio *q*()/*q*(*x*), and  is the determinant of the Jacobian resulting from the potential change of dimension of the parameter vector.

Accordingly, for the application of MCMC to infer the functional form of demographic history a variety of components need to be specified:

• a suitable *parameterization *of the estimated function *N*_*e*_(*t*)

• the *likelihood function*,

• a *prior distribution *for each considered variable, and,

• rules to construct the *Markov chain *(i.e. acceptance probabilities).

In the following sections we now describe each of these elements in detail. For further general information on the statistical and mathematical background of the MCMC algorithm we refer to the many excellent monographs on this topic (e.g., [25]).

#### Parameterization of *N*_*e*_(*t*)

In our suggested procedure we approximate the sampled demographic history *N*_*e*_(*t*) by a piecewise linear function. This spline of first order degree consists of a first node at position *a*_0 _= 0 and height *h*_0_, followed by *k *internal supporting nodes at (*a*_1_; *h*_1_), (*a*_2_;*h*_2_),..., (*a*_*k*_; *h*_*k*_), and a terminal node at  with height *h*_*k*+1 _Hence, the spline is defined for all *t *∈ [0, *T*], and for any given *k *the it contains *k *free position parameters and *k *+ 2 free height parameters. Note that, unlike in the skyline plot, we do not constrain the change-points *a*_1_,..., *a*_*k *_to lie on the grid points defined by the inter-node distances *w*_*i*_. Moreover, we also allow that the number of internal nodes *k *changes during sampling of the population function from the posterior. Hence, *k *is technically a hyper-parameter that controls the roughness of the resulting spline. As will be clear from the outline of the MCMC algorithm below, note that the final point estimate  obtained from posterior sampling will be a mixture of linear splines (i.e. a smooth and possibly nonlinear function) rather than a single spline.

#### Likelihood function

The likelihood *L *employed in our procedure is the product of the densities of the waiting times between subsequent coalescence events, i.e. . This function depends via Eq. 2 on the effective population size *N*_*e*_(*t*), and hence indirectly on the spline parameters *a*_*i*_, *h*_*i *_and *k*. Because *N*_*e*_(*t*) is represented by a linear spline, calculation of the likelihood can be done in a computationally efficient fashion.

#### Prior distributions

##### Number of change-points

Following [18] we employ a truncated Poisson-distribution as the prior distribution for *k*, i.e.



where *c *is a normalizing constant to ensure that P(*k*) is a proper distribution. For the hard upper limit of the number of change-points we use *k*_max _= 30. The parameter *λ *acts as a smoothing parameter, set in a typical analysis to about *λ *= 0.1 - 1.0.

As an alternative to using a fixed *λ *we also suggest a hierarchical Bayes approach where *λ *is drawn from a Gamma distribution



with some shape parameter *a *and scale parameter *b *(for instance, *a *≈ 0.5 and *b *≈ 2 so that E(*λ*) = *ab *≈ 1 and Var(*λ*) = *ab*^2 ^≈ 2).

##### Positions

We assume that the internal nodes of the spline are *a priori *uniformly distributed in the interval [0, *T*]. As a simple trick to avoid very small inter-node distance we generate *2k *+ 1 random variables, and set the change-points *a*_*j *_= *z*_[2*j*] _for *j *= 1,..., *k*. The corresponding joint density is



with *a*_0 _= 0 and *a*_*k*+1 _= *T*.

##### Heights

As prior distributions for the heights *h*_*i *_we assume a Gamma distribution

Gamma(*h*_*i*_|*α*_*i*_, *β*_*i*_)     (9)

which ensures that sampled heights are always positive. The parameters *α*_*i *_and *β*_*i *_determine the *a priori *mean and variance of height *h*_*i*_. More generally, one can also allow fully time-dependent prior parameters *α*(*t*) and *β*(*t*). This is particularly advisable if the population size is known in advance to be subject to large changes in time.

In a strict Bayesian approach, the choice of the prior distribution for the heights is completely external to the observed data. One simple possibility would, e.g., be to assume an arbitrary constant for the mean and variance. However, we recommend to follow a more pragmatic "empirical Bayes" route and to use the data at hand (or some other related data set) to obtain an informed guess about the prior heights. For example, an assumed constant population size as prior mean could be estimated using the method by Felsenstein [13]. Another possibility is to employ the skyline plot as a prior mean estimate (this is the default in our program).

However, note that in practice the actual choice of prior height distribution seems to matter only little for estimating the posterior demographic function (see Figure 2 and the section on simulated data below). Only when there are few coalescent events per unit of time will the posterior estimate of the demographic function be dominated by the prior.

#### Construction of the Markov chain

There are four different possibilities to change the state defined by the parameters *c*_*i*_, *h*_*i*_, and *k *of the spline describing the effective population size *N*_*e*_(*t*):

1. varying the position of a change-point (i.e. internal node),

2. changing the height at a certain change-point,

3. generating a new change-point ("birth" step), and

4. deleting an existent change-point ("death" step).

Let *η*_*k*_, *π*_*k*_, *b*_*k*_, and *d*_*k *_the probabilities of the four moves given *k*, with *η*_*k *_+ *π*_*k *_+ *b*_*k *_+ *d*_*k *_= 1. In order to satisfy the requirement of detailed balance in the corresponding Markov chain the probabilities of birth and death steps (*b*_*k *_and *d*_*k*_) need to be synchronized [18]. This can be achieved, e.g., by setting



and



where *c *is chosen so that *b*_*k *_+ *d*_*k *_< 0.9 for all *k*.

Next, we describe the individual procedures to propose and accept one of the above four moves as implemented in our program.

##### Height change

First, a height *h*_*j *_is selected out of the *k *+ 2 existing heights with probability . Second, a new height  is generated by  = *h*_*j *_exp(*z*), where *z *is a uniformly distributed random variable on . Third, the new height is accepted with probability



where *α *and *β *are from the prior distribution and  denotes the ratio of the likelihood of the new state  (with modified height) and the likelihood of the current state *x*.

##### Position move

First, a change-point *a*_*j *_is chosen randomly with probability . Second, its new position  within [*a*_*j*-1_, *a*_*j*+1_] is determined by drawing from the corresponding uniform distribution. Third,  is accepted with probability



##### Birth step

First, the position a* of the new change-point is found by uniformly drawing from (0, *L*), and let the neighboring nodes left and right of a* have positions *a*_*j *_and *a*_*j*+1_. Second, the corresponding new height h* is generated by randomly disturbing the current height *N*_*e*_(a*) on the position a* according to *N*_*e*_(a*) + *zN*_*e*_(a*) where *z *is a uniformly distributed random variable on the interval . Note that the birth step increases the dimension of the parameter vector from 2*k *+ 2 to 2*k *+ 4 as a new change-point and a new height are generated.

The corresponding acceptance probability of the birth step is computed according to Eq. 5 with likelihood and prior ratios as above, and with proposal ratio



and Jacobi determinant



##### Death step

This is the inversion of the birth step and consists of removing a change-point. First, a* chosen from *a*_1_,..., *a*_*k *_with probability . Second, the corresponding height h* is also removed from the vector of spline parameters. The acceptance probability for the death step is



where the proposal ratio and the Jacobi determinant is the same as for the birth step.

#### Computation of estimated *N*_*e*_(*t*) and associated confidence intervals

In order to obtain an estimate of the effective population size in time we now proceed as follows. First, the Markov chain is started with an initial state that corresponds to a completely flat demographic function, i.e. *N*_*e*_(*t*) = *c*, where c is some rough estimate of population size, and *k *= 0. Second, 100,000 repeats of the MCMC algorithm are performed, of which the first 5,000 are ignored to allow for a "burn-in" period.

Third, the remaining samples are thinned out by a factor of 1:50 to remove auto-correlation. As a result, 1900 independent samples from the joint posterior of the spline parameters *a*_*i*_, *h*_*i *_and *k *are obtained.

Subsequently, in order to obtain a point estimate  and associated confidence bands we compute the distribution of the effective population size at a number of fixed equidistant time points *t*_1_, *t*_2_,..., *t*_1000 _∈ [0, *T*]. Finally, we report as summary statistics the corresponding median and the lower and upper 2.5% quantiles.

#### Extension to multiple genealogies

In this paper we have introduced non-parametric sampling of demographic histories assuming a fixed underlying genealogical tree (or equivalently, a fixed set of inter-node distances *w*_*n*_, *w*_*n*-1_,..., *w*_2_.)

However, in our approach – unlike previous non-parametric methods such as the skyline plot – it is also conceptually straightforward to incorporate phylogenetic error.

This can be done by joint sampling of trees and demographies according to the following simple algorithm:

1. Given sequence data *D*, sample a tree G* with clock-like branch lengths (see, e.g, refs. [8,9,11,12,26] for suitable methods).

2. Use the method described in this paper to sample the demographic function conditioned on the inter-node distances  from G*.

3. Repeat steps 1 and 2 to obtain the posterior distribution for the population size function, now conditioned on *D *rather than on some given *w*_*n*_, *w*_*n*-1_,..., *w*_2_.

Note that each sampled tree may have a different depth . This means that the interval [0, *T*] for the prior (and posterior) height distribution has to be set in advance (and independent of the T*). For the case of 0 <*t *<T* sampling of heights then proceeds as described above, while for T* <*t *<*T *– the region with no data from a given sampled tree – the heights are simply drawn from the respective prior distribution.

## Discussion

In order to test the potential of the proposed reversible jump MCMC algorithm we first applied it to synthetic data simulated according to various demographic scenarios. Subsequently, we reanalyzed two viral data sets from Central Africa and Egypt.

### Computer program

The proposed framework has been implemented by us for the case of a single underlying genealogy. The program is written in the statistical computer language R [19] and is incorporated in recent versions of the R package APE [20].

To install the APE package, simply run the R program, and enter at the R prompt

install.packages("ape")

This downloads the APE package from the Internet. The proposed reversible jump MCMC approach is implemented in the function "mcmc.popsize" of which an extensive description along with examples can be obtained online by typing

library("ape")

help(mcmc.popsize)

into the R command window. The APE package also includes routines for plotting the inferred population function (e.g., all figures in this paper were prepared with APE).

Note that the use of this R program is only valid if the phylogenetic error is low – this is typically the case when the evolutionary rate is high and the available sequences are long (e.g. viral data). If the phylogenetic error is not negligible compared to the coalescent error, please use software such as BEAST [27].

### Simulated data

In the simulation setup we followed Pybus et al. [14] and Strimmer and Pybus [17]. Specifically, we performed simulations assuming constant population size (*N*_*e*_(*t*) = 100) as well as exponential population growth (*N*_*e*_(*t*) = l000*e*^-*t*^), using 25 and 100 sampled lineages, respectively. To estimate the population size function we employed the proposed MCMC algorithm and the classic and generalized skyline plot. In the former the smoothing parameter *λ *was drawn from the hierarchical model with default parameters (*a *= 0.5 and *b *= 2).

**Figure 1:**
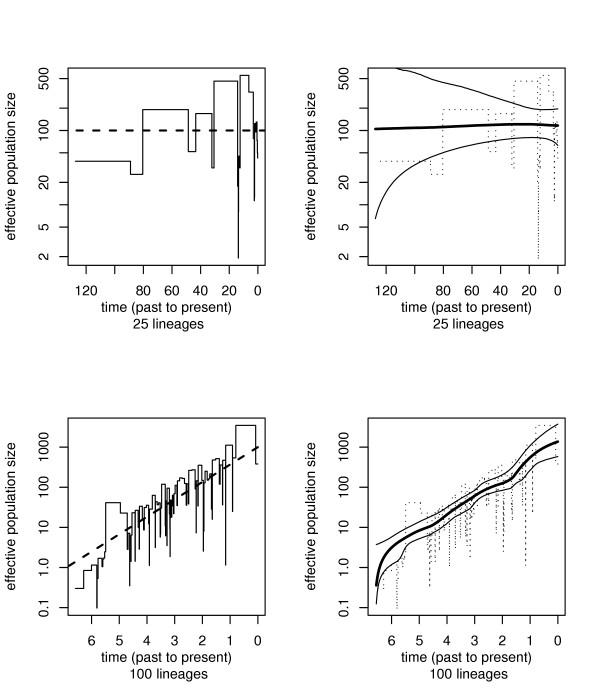
**Simulated data ***Top row*: Example of a simulation with constant population size: (left) true demographic history (dashed line) and estimate obtained with the classic skyline plot; (right) point estimate obtained with rjMCMC and 95% confidence band. *Bottom row*: Example with exponential population growth: (left) true population growth and classic skyline plot; (right) results from rjMCMC approach.

Figure 1 shows the results from a typical run of the simulations. The top row illustrates the case of constant population size, whereas the bottom row demonstrates exponential growth. On the left in Figure 1, top row, the true underlying constant population size is shown (the thick dashed line), together with the estimate provided by the classic skyline plot. On the right, this is contrasted with the estimate obtained by using our reversible jump MCMC algorithm. Clearly, the median of the posterior distribution of *N*_*e*_(*t*) is a very good point estimator of the true demographic history. In addition, the 95% confidence band is also automatically obtained by the MCMC method. Interestingly, it can be immediately seen that the uncertainty in *N*_*e*_(*t*) increases with a growing distance from the present. This simply reflects the fact that near the root of the tree for constant population size there are only few coalescent events.

In Figure 1, bottom row, an example for a simulation with an exponentially growing population is shown. As for the constant population, the rjMCMC algorithm is capable of recovering the original population size function (shown as thick dashed line) complete with confidence bands, whereas the skyline plot contains a large amount of stochastic noise, and only provides a rough exploratory picture of the population size changes.

In Figure 2 the influence of the choice of prior demographic function on the final posterior estimate is investigated using further simulations of an exponentially growing population. The left column depicts the prior distributions (specifically the 2.5%, 50% and 97.5% quantiles for each time point) for two typical cases: a constant prior function (= constant population size with constant variance), and the "skyline plot" prior function (= time dependent piecewise- constant population size and variance). The right column of figure 2 presents the corresponding posterior distributions as obtained with the present rjMCMC approach. The results for both cases are very similar. This indicates that there is sufficient signal in the data to make the posterior demographic function (almost) independent from the choice of prior distribution. Note that only near the left and right end of the investigated time intervals there are some slight differences. These can be explained by the lack of data points near the borders.

### HIV-1 in Central Africa

Next, we applied our method to infer the demographic history from a set of HIV-1 sequences from Central Africa. These data was originally used by Vidal et al. [28] who examined the genetic diversity of HIV-1 type M in this region. Further detailed analysis can be found in Rambaut et al. [29] and Yusim et al. [30]. Here we use the reconstructed phylogeny of Yusim et al. with which Strimmer and Pybus also estimated the demographic history by means of the generalized skyline plot [17].

**Figure 3:**
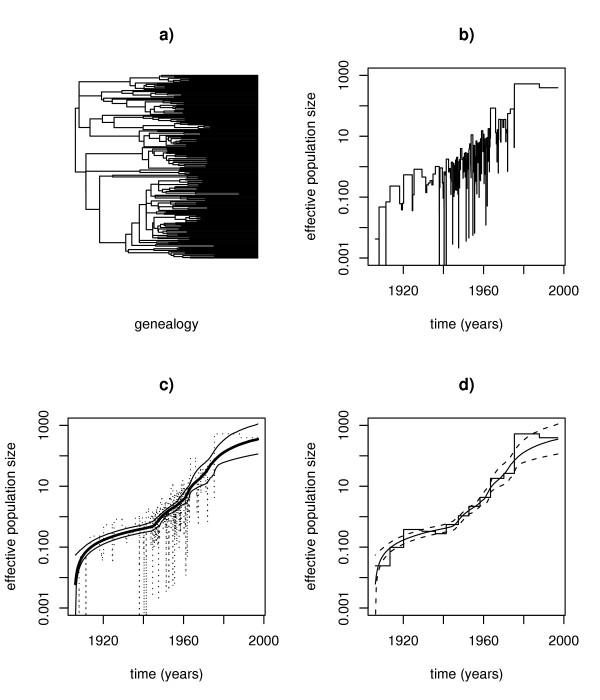
**HIV-1 in Central Africa ***Top row*: a) underlying genealogy; b) classic skyline plot. *Bottom row*: c) population size function estimated with rjMCMC and corresponding 95% confidence band; d) comparison rjMCMC versus generalized skyline plot.

Figure 3 shows the result of the analysis with the reversible jump MCMC algorithm compared with the predictions from the classic and generalized skyline plots. As in Yusim et al. [30] an evolutionary rate of 0.0023 substitutions per year was assumed to convert the time axis into units of years. The first row of Figure 3 displays the tree of Yusim et al. [30] and the corresponding classic skyline plot. The latter exhibits a large amount of noise, nevertheless the main demographic signal is clearly visible in the graph. In contrast, in Figure 3c (second row) the effective population size as estimated by the rjMCMC algorithm is displayed. The thick line shows the median and the thin lines the 95% confidence interval. Especially in the middle part of the figure, where most of the data is located, the confidence interval is very narrow, indicating a stable estimation of the demographic history. Also note that for this data the average number of change-points in the MCMC run was *k *= 9.25, i.e. the estimated effective degree of freedom is much less than that implicitly assumed in the classic skyline plot.

A comparison with the generalized skyline plot [17] is shown in Figure 3d. This demonstrates that the generalized skyline plot, in contrast to its classic cousin, provides a very good noise-reduced approximation to the demographic history as estimated by the reversible jump MCMC approach. However, especially near the present the step function employed in the generalized skyline plot leads to unrealistic jumps in the population size that are not present in the smooth estimate provided by the proposed MCMC method.

### HCV in Egypt

In Egypt 10%-20% of the general population are infected with the Hepatitis C virus (HCV) [31]. This endemicity seems mainly to be caused by percutaneous medical procedures such as needle injections that took place during a countrywide health campaign between 1964 and 1982 against schistosomiasis. In order to investigate this phenomenon blood samples were obtained from various regions of Egypt and used to study the epidemic history of Hepatitis C. For instance, Tanaka et al. [32] analyzed the molecular evolution of HCV genotype 4a. Specifically, they utilized 47 sequences (AF217800-AF217812 [31] and AB103424-AB103457 [32]) from the NS5B region of the HCV subtype 4a to reconstruct the respective phylogeny, and subsequently applied the skyline plot method to infer the demographic history.

**Figure 4:**
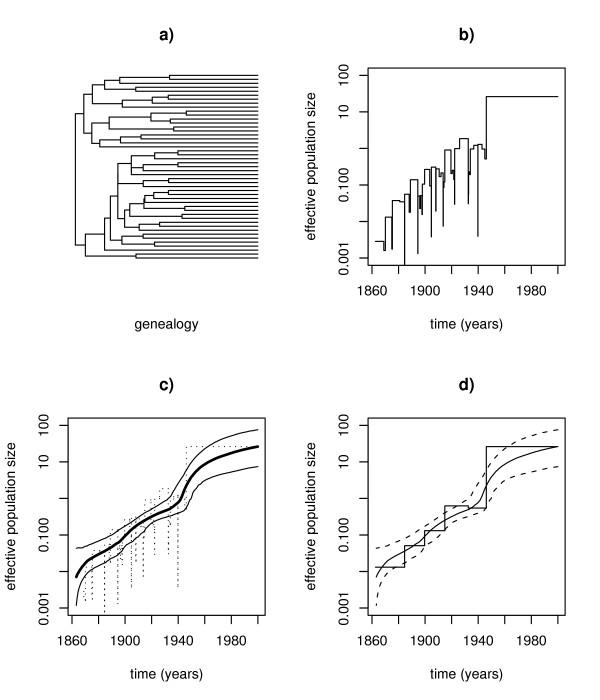
**HCV in Egypt ***Top row*: a) underlying reconstructed genealogy; b) classic skyline plot. *Bottom row*: c) population size function estimated with rjMCMC and corresponding 95% confidence band; d) comparison rjMCMC versus generalized skyline plot.

We repeated their analysis with the reversible jump MCMC approach developed in this paper. We downloaded the sequence data from the HCV sequence database [33] and inferred the corresponding maximum-likelihood genealogy using the TREEFINDER program [34]. This tree is depicted in Figure 4a, next to the demographic history estimated from it by the classic skyline plot (Figure 4b). In the bottom of the figure we show the estimated population size function and its 95% confidence bands as obtained by our rjMCMC method (Figure 4c) and we also compare our results with those of the generalized skyline plot (Figure 4d). For the generating the time axis in these plots we assumed an evolutionary rate of 0.00045 substitutions per year.

Generally, the star-like shape of the inferred tree already is indicative of exponential growth. This is confirmed by both the skyline plot as well as by our analysis (Figure 4d). Moreover, it can be seen that around 1940 the growth rate increased (i.e. the slope of *N*_*e*_(*t*) in the log-plot changes). Near the present, the rate decreased again. Also note the broadening of the confidence interval since 1940 which reflects the sparsity of available observations. This implies that the claim of Tanaka et al. [32] that the demographic history recently changed back to constant population size after an exponential growth is not firmly backed by the data. For further biological analysis of the HCV data we refer to Pybus et al. [35].

## Conclusions

We have presented a new approach to non-parametric inference of demographic history from an inferred genealogy. This method is based on reversible jump MCMC sampling of the population size function *N*_*e*_(*t*). Unlike its predecessors, the classic and generalized skyline plots, it returns a smooth and realistic estimate of the demographic history and thus overcomes the constraints due to assuming a step function. Moreover, it automatically provides confidence limits. Nevertheless, the procedure is still computationally fast and can be run on any standard PC hardware.

In our examples we demonstrated the advantage of non-parametric estimation of demographic history. Parametric estimation always assumes a certain functional form of population growth which may lead to problematic statements (cf. the HCV data set), in particular if the confidence bands of the estimated function *N*_*e*_(*t*) are not taken into account.

From the methodological point of view, model selection via rjMCMC has the advantage that the effective dimension, i.e. the degree of smoothing, is automatically chosen in a data-driven manner. There is only one parameter (*λ*) that controls the *a priori *degree of smoothing, and this is adjusted accordingly by the investigated data. In addition, a further advantage of our MCMC approach is that – in contrast to the skyline plot – at least in principle it is straightforward to incorporate it in more general MCMC sampling schemes that also take account of the uncertainty in the genealogy.

During the referee process we have learned that the authors of the software package BEAST [27] have developed a similar non-parametric method to Bayesian coalescent inference of population history (A.J. Drummond et al., in preparation). We plan to work with Dr. Drummond to make available in BEAST joint sampling of sampling of demographic histories and of trees. This would combine the present rjMCMC approach and the method developed by Drummond and colleagues.

## Authors' contributions

This paper summarizes the main results from a master's thesis of R.O. supervised by K.S. and L.F. Accordingly, K.S. and L.F. jointly devised the project and R.O. carried out all analyses and simulations. All authors participated in the development of methodology. R.O. and K.S. wrote the manuscript. All authors approved of the final version.
